# Electrocardiogram analysis in Anderson-Fabry disease: a valuable tool for progressive phenotypic expression tracking

**DOI:** 10.3389/fcvm.2023.1184361

**Published:** 2023-06-21

**Authors:** V. Parisi, R. Baldassarre, V. Ferrara, R. Ditaranto, F. Barlocco, R. Lillo, F. Re, G. Marchi, C. Chiti, F. Di Nicola, C. Catalano, L. Barile, M. A. Schiavo, A. Ponziani, G. Saturi, A. G. Caponetti, A. Berardini, M. Graziosi, F. Pasquale, I. Salamon, M. Ferracin, E. Nardi, I. Capelli, D. Girelli, J. R. Gimeno Blanes, M. Biffi, N. Galiè, I. Olivotto, F. Graziani, E. Biagini

**Affiliations:** ^1^ Cardiology Unit, Cardiac Thoracic and Vascular Department, IRCCS Azienda Ospedaliero-Universitaria di Bologna, Bologna, Italy; ^2^ Department of Medical and Surgical Sciences (DIMEC), University of Bologna, Bologna, Italy; ^3^ Unità Ospedaliera Cardiologia, Azienda Sanitaria Territoriale Pesaro Urbino, Fano, Italy; ^4^ Department of Experimental and Clinical Medicine, Careggi University Hospital, University of Florence, Florence, Italy; ^5^ Department of Cardiovascular Sciences, Fondazione Policlinico Universitario A. Gemelli IRCCS, Rome, Italy; ^6^ Cardiology Department, San Camillo-Forlanini Hospital, Rome, Italy; ^7^ Internal Medicine Unit and MetabERN Health Care Provider, Azienda Ospedaliera Universitaria Integrata di Verona, Verona, Italy; ^8^ European Reference Network for Rare, Low Prevalence and Complex Diseases of the Heart-ERN GUARD-Heart, Bologn, Italy; ^9^ Nephrology, Dialysis and Renal Transplant Unit, IRCCS, Azienda Ospedaliero-Universitaria di Bologna, Bologna, Italy; ^10^ European Rare Kidney Disease Reference Network-ERKNet, Bologna, Italy; ^11^ Inherited Cardiac Disease Unit, University Hospital Virgen de la Arrixaca, Murcia, Spain; ^12^ Department of Experimental and Clinical Medicine, University of Florence, Meyer University Children Hospital and Careggi University Hospital, Florence, Italy

**Keywords:** Anderson-Fabry disease, cardiac involvement, left ventricular hyperertrophy, electrocardiogram (ECG), bundle branch block, repolarization abnormalities, ECG pattern

## Abstract

**Background:**

Electrocardiogram (ECG) has proven to be useful for early detection of cardiac involvement in Anderson-Fabry disease (AFD); however, little evidence is available on the association between ECG alterations and the progression of the disease.

**Aim and Methods:**

To perform a cross sectional comparison of ECG abnormalities throughout different left ventricular hypertrophy (LVH) severity subgroups, providing ECG patterns specific of the progressive AFD stages. 189 AFD patients from a multicenter cohort underwent comprehensive ECG analysis, echocardiography, and clinical evaluation.

**Results:**

The study cohort (39% males, median age 47 years, 68% classical AFD) was divided into 4 groups according to different degree of left ventricular (LV) thickness: group A ≤ 9 mm (*n* = 52, 28%); group B 10–14 mm (*n* = 76, 40%); group C 15–19 mm (*n* = 46, 24%); group D ≥ 20 mm (*n* = 15, 8%). The most frequent conduction delay was right bundle branch block (RBBB), incomplete in groups B and C (20%,22%) and complete RBBB in group D (54%, *p* < 0.001); none of the patients had left bundle branch block (LBBB). Left anterior fascicular block, LVH criteria, negative T waves, ST depression were more common in the advanced stages of the disease (*p* < 0.001). Summarizing our results, we suggested ECG patterns representative of the different AFD stages as assessed by the increases in LV thickness over time (Central Figure). Patients from group A showed mostly a normal ECG (77%) or minor anomalies like LVH criteria (8%) and delta wave/slurred QR onset + borderline PR (8%). Differently, patients from groups B and C exhibited more heterogeneous ECG patterns: LVH (17%; 7% respectively); LVH + LV strain (9%; 17%); incomplete RBBB + repolarization abnormalities (8%; 9%), more frequently associated with LVH criteria in group C than B (8%; 15%). Finally, patients from group D showed very peculiar ECG patterns, represented by complete RBBB + LVH and repolarization abnormalities (40%), sometimes associated with QRS fragmentation (13%).

**Conclusions:**

ECG is a sensitive tool for early identification and long-term monitoring of cardiac involvement in patients with AFD, providing “instantaneous pictures” along the natural history of AFD. Whether ECG changes may be associated with clinical events remains to be determined.

## 1. Introduction

Anderson-Fabry disease (AFD) is an X-linked lysosomal storage disorder, caused by GLA gene mutations which lead to a reduction in *α-galactosidase A* enzyme activity (1, 2). The result is the accumulation of lysosomal globotriaosylceramide (Gb3) and related globotriaosylsphingosine (lysoGb3) in many tissues, including the heart, kidneys, vessels, and peripheral nervous system (3). The heart is frequently involved, both in the classical multisystemic disease and in the so-called “late-onset” variant (a predominantly cardiac disease that generally occurs after the third decade of life), and cardiovascular involvement is the main cause of mortality (4). Although our understanding of the pathophysiological mechanisms and the natural history of the disease have greatly increased in recent years, together with improved therapeutic options, there are still several open issues potentially leading to diagnostic delay and thus impacting on the long-term prognosis. In recent years, 12-lead electrocardiogram (ECG) analysis has acquired considerable importance as a valuable tool in the management of AFD patients, given its widespread availability, its easy acquisition, and its negligible cost. Many papers (5–8) have been published describing the typical ECG pattern of AFD and our group have recently demonstrated how some of these peculiar electrocardiographic features can help to differentiate AFD from HCM while investigating unexplained left ventricular hypertrophy (9); however, studies aiming to establish an association between the temporal evolution of ECG patterns with progressive cardiac involvement are lacking. In the present study we therefore performed a cross-sectional comparison of ECG abnormalities across subgroups of increasing severity of left ventricular hypertrophy (LVH), with the hypothesis that the tracing might provide a reliable estimation of the underlying disease stage.

## 2. Materials and methods

In this retrospective, international, multicenter cohort study 215 patients with AFD from six Centres were evaluated: Cardiology Unit, St. Orsola Hospital, IRCCS Azienda Ospedaliero-Universitaria of Bologna; Azienda Ospedaliero-Universitaria Careggi, Florence; Policlinico Universitario Agostino Gemelli, Rome; Azienda Ospedaliera San Camillo, Rome; Azienda Ospedaliera Integrata, Verona; Hospital Universitario Virgen de la Arrixaca Ctra, Murcia AFD diagnosis was based on the measurement of *α-galactosidase A* enzyme activity in leucocytes (in male patients) and/or plasmatic lyso-Gb3 levels with the dried blood spot method. The diagnosis was confirmed by genetic sequencing of the GLA gene*.* For each patient main clinical, echocardiographic, and 12-lead ECG data recorded at the first evaluation in each participation center were collected. Records of the first evaluation were revised to extract the following data: clinical characteristics (age, gender, age at diagnosis, classic/late onset form, systemic involvement, cardiologic and AFD specific therapy); main echocardiographic findings (LV diameters; left ventricle ejection fraction (LVEF), left atrium diameter (LAD); left ventricle outflow tract obstruction (LVOT) defined as > defined as an instantaneous peak Doppler LV outflow tract pressure gradient ≥30 mmHg at rest or during physiological provocation such as Valsalva maneuver, standing and exercise (10)). The ECG analysis protocol was performed as stated below. In accordance with previous outcome studies on hypertrophic cardiomyopathy (HCM) [9], data were analyzed in these 4 LVWT subgroups: group A, ≤9 mm; group B, 10–14 mm; group C, 15–19 mm; group D, ≥20 mm. For each group, ECG characteristics were classified, and patterns representative of different AFD stages were identified. The study was approved by the local Ethics Committee of the participating centers and was conducted in accordance with the principles of the most recent revision of the Declaration of Helsinki.

### 2.1. ECG analysis

The 12-lead ECG (standard calibration of 10 mm/1 mV and normal paper speed of 25 mm/s) recorded in the supine position was independently analyzed at IRCCS University Sant' Orsola Hospital of Bologna by three different investigators (V.F., R.B., F.D.N.); discrepancies were solved by three senior supervisors (R.D., E.B., M.B.). For all patients, classical ECG parameters were collected: heart rate, RR interval, PR interval, QRS complex duration, QT, and corrected QT (QTc) with Bazett's formula. In patients with atrial fibrillation, the arithmetical average of the RR interval was recorded. PR interval was measured from the beginning of the P wave to the first QRS deflection and was classified as normal (120–200 ms—and specified as borderline if 120–130 ms), short (<120 ms); first-degree atrioventricular (AVB) was diagnosed if PR ≥ 200 ms. P wave duration and P wave end-to-Q wave interval in DII were also specified. Corrected QT (Bazett's formula) was considered pathological if ≥ 450 ms in males and ≥ 470 ms in females. Intraventricular conduction delay (complete/incomplete right bundle branch block (RBBB), left bundle branch block (LBBB), left anterior fascicular block (LAFB), non-specific intraventricular conduction delay) were defined as previously stated (11). Left/right atrial enlargement and left/right axis deviation were considered as dichotomous variables. Left ventricular hypertrophy (LVH) was defined at the ECG by at least one of the following criteria: Sokolow-Lyon index (SV1 or SV2 + RV5 or RV6 ≥ 3.5 mV); Cornell index (SV3 + R aVL ≥ 2.0 mV in females and ≥ 2.8 mV in males); R wave amplitude in aVL ≥ 1.1 mV. Patients with a Sokolow-Lyon index ≥ 50 mm were classified as having massive LVH. Total QRS score was defined by the sum of zenith-to-nadir QRS amplitudes of all 12 leads. Other specific ECG characteristics were evaluated: pseudonecrosis (Q wave deeper than 1/3 of the R wave, and/or Q wave ≥ 40 ms in 2 contiguous derivations except aVR, and/or absence of R wave amplitude progression in precordial leads); low QRS voltages (amplitude < 0.5 mV in all DI, DII, and DIII); fragmented QRS complex (RsR' pattern ≤ 120 ms in two contiguous leads, and/or R/S waves notching); T wave alterations (negative or positive if amplitude ≥ 0.1 mV, giant negative or positive if amplitude ≥ 1 mV); slurred QRS onset associated with borderline PR interval. Maximum T wave amplitude was measured from the isoelectric line to the apex (either negative or positive).

## 3. Statistical analysis

Categorical variables are expressed as frequencies and percentage; continuous data were expressed as median and IQR. Comparisons among the four groups were performed with Pearson's chi-squared test for categorical variables and using Kruskal Wallis test for continuous data. A *p* value ≤ 0.05 was considered statistically significant.

## 4. Results

Six patients with poor ECG quality, 3 patients with no LVWT data, and 17 patients ≤ 18 years old were excluded; none of the patient had a paced ventricular rhythm. The final cohort study was then composed of 189 AFD patients, distributed as follows within the different LVWT subgroups: group A, *n* = 52 (28%); group B, *n* = 76 (40%); group C, *n* = 46 (24%); group D, *n* = 15 (8%).

Table 1 shows the baseline clinical and echocardiographic characteristics of study population. Of all the189 patients, 73 (39%) were males, with a median age of 47 years old (IQR 35–58). AFD diagnosis was done by family screening programs (76% of the entire cohort), or systemic manifestations: 9% cardiac, 7% renal, 5% neurologic, 2% ophthalmologic, 1% dermatologic. The classical AFD phenotype was present in 128 patients (68%), while 61 (32%) had a late-onset phenotype.

**Table 1:** Baseline clinical and echocardiographic characteristics of the study population.

	Overall (*N* = 189)	Group A (*N* = 52)	Group B (*N* = 76)	Group C (*N* = 46)	Group D (*N* = 15)	*p*-value
*Male sex*	73 (39%)	6 (11%)	32 (42%)	23 (50%)	12 (80%)	<0.001
*Age at first evaluation (years)*	47 (35–58)	33 (25–42)	45 (36–57)	57 (49–67)	53 (49–61)	<0.001
AFD phenotype						
*Classic phenotype*	128 (68%)	33 (63%)	53 (70%)	31 (67%)	11 (73%)	0.606
*Late onset phenotype*	61 (32%)	19 (36%)	23 (30%)	15 (33%)	4 (27%)	0.606
*Hypertension* (*N* = 187)	64 (34%)	4 (8%)	29 (38%)	19 (42%)	12 (80%)	<0.001
Heart Rhythm						
*Sinus rhythm*	172 (91%)	52 (100%)	68 (89%)	40 (87%)	12 (80%)	0.01
*Atrial fibrillation/flutter*	13 (7%)	0	6 (8%)	5 (11%)	2 (13%)	
*Positive family history for SCD* (*N* = 87)	3 (3%)	0	1 (2%)	2 (6%)	0	0.393
*Ocular involvement* (*N* = 135)	51 (27%)	15 (29%)	18 (24%)	12 (26%)	6 (40%)	0.048
Neurological involvement (*N* = 175)						
*TIA*	17 (10%)	4 (8%)	7 (10%)	3 (7%)	3 (23%)	0.162
*Ischemic stroke*	2 (1%)	0	0	2 (5%)	0	
*Angiokeratomas* (*N* = 144)	44 (30%)	8 (23%)	20 (33%)	12 (33%)	4 (31%)	0.785
*Acroparaesthesia* (*N* = 160)	59 (37%)	16 (36%)	29 (45%)	11 (28%)	3 (23%)	0.232
*Gastrointestinal symptoms* (*N* = 142)	40 (28%)	14 (40%)	14 (24%)	9 (25%)	3 (25%)	0.357
*Dialysis* (*N* = 185)	9 (5%)	0	3 (4%)	4 (9%)	2 (14%)	0.72
*Kidney transplantation* (*N* = 183)	10 (5%)	0	1 (1%)	3 (7%)	6 (40%)	<0.001
*eGFR (ml/min)*	94 (76–112)	112 (92–118)	99 (79–115)	75 (62–84)	68 (48–106)	<0.001
Cardiologic therapy (*N* = 104)						
*Antithrombotic therapy*	34 (18%)	2 (7%)	11 (27%)	15 (59%)	6 (75%)	<0.001
*Beta-blocker*	28 (27%)	1 (4%)	5 (12%)	16 (59%)	6 (75%)	
*Calcium antagonist*	4 (4%)	0	3 (7%)	1 (4%)	0	
*Anti-arrhythmic drugs*	3 (3%)	0	2 (6%)	1 (4%)	0	
AFD specific therapy (*N* = 174)						
*Alpha-agalsidase*	79 (45%)	18 (39%)	35 (49%)	17 (39%)	9 (64%)	0.01
*Beta-agalsidase*	32 (18%)	5 (11%)	11 (15%)	13 (30%)	3 (21%)	
*Chaperone therapy*	14 (8%)	2 (4%)	5 (7%)	7 (16%)	0	
Echocardiogram						
*LVEDD (mm)*	45 (43–48)	43 (42–46)	46 (44–49)	45 (41–48)	47 (42–49)	0.019
*LVEDV (ml)*	83 (73–105)	77 (72–86)	88 (77–106)	91 (68–110)	73 (64–115)	0.146
*LVEF (%)*	64 (60–68)	65 (62–69)	64 (60–67)	63 (59–68)	59 (56–67)	0.066
*LV mass (g/m2)*	115 (79–143)	67 (62–72)	111 (91–123)	156 (121–177)	203 (158–283)	<0.001
*LAD (mm)*	36 (31–41)	30 (29–34)	36 (33–40)	41 (36–45)	48 (44–51)	<0.001
*LVOT obstruction (N = 166)*	3 (2%)	0	0	1 (3%)	2 (15%)	
Maximal LVWT localization at echo (*N* = 87)						
*Interventricular septum*	70 (80%)	12 (92%)	21 (70%)	27 (79%)	10 (100%)	0.24
*Postero-lateral wall*	12 (14%)	1 (8%)	8 (27%)	3 (9%)	0	
*Apex*	3 (3%)	0	1 (3%)	2 (6%)	0	
*Symmetric LVH*	2 (2%)	0	0	2 (6%)	0	

AFD, anderson fabry disease; eGFR, estimated glomerular filtration rate with CKD-EPI (chronic kidney disease epidemiology collaboration) formula; ICD, implanted cardioverter defibrillator; LAD, left atrial diameter (in parasternal long axis view); LV, left ventricle; LVEDD, left ventricular end diastolic diameter; LVEDV, left ventricular end diastolic volume; LVH, left ventricular hypertrophy; LVOT, left ventricular outflow tract; LVWT, left ventricular wall thickness; NSVT, non sustained ventricular tachycardia; PM, pacemaker; SCD, sudden cardiac death; TIA, transient ischemic attack.

Overall, median LVWT was 13 mm (IQR 9–16), mostly localized at the interventricular septum (80%); median LV ejection fraction was 64% (IQR 60–68); 4 patients had an implantable cardioverter defibrillator (ICD), 3 patients had a pacemaker (PM).

125 patients (66%) were treated with specific AFD therapy: 111 were on enzyme replacement therapy (79 on recombinant alpha-agalsidase and 32 on beta-agalsidase A); 14 were on chaperone therapy with migalastat.

Table 2 summarizes the 12-leads ECG characteristics according to the 4 different study groups.

**Table 2:** ECG features of the study population according to the different groups.

	Overall (*N* = 189)	Group A (*N* = 52)	Group B (*N* = 76)	Group C (*N* = 46)	Group D (*N* = 15)	*p*-value
*Pathologic ECG*	97 (51%)	7 (13%)	35 (46%)	41 (89%)	14 (93%)	<0.001
*Heart rate (bpm)*	67 (60–78)	74 (60–83)	67 (60–78)	63 (54–69)	65 (60–78)	0.021
*RR interval (ms)*	880 (800–1,000)	800 (720–996)	880 (794–1,000)	975 (800–1,090)	900 (800–1,000)	0.024
*Sinus rhythm*	180 (95%)	52 (100%)	72 (95%)	43 (93%)	13 (87%)	0.582
*Atrial fibrillation/flutter*	9 (5%)	0	4 (5%)	3 (6%)	2 (13%)	0.364
*PR interval (ms)*	143 (128–160)	137 (125–154)	140 (127–160)	160 (130–180)	170 (130–180)	0.002
*PR interval < 120 ms (N = 180)*	16 (9%)	7 (13%)	7 (10%)	2 (5%)	0	0.278
*Borderline PR interval (N = 175)*	46 (26%)	14 (27%)	19 (27%)	9 (22%)	4 (31%)	0.881
*P wave DII (ms)*	80 (80–100)	80 (80–90)	80 (80–94)	100 (80–110)	80 (80–120)	0.001
*PQ interval (ms)*	50 (40–70)	55 (40–70)	54 (40–67)	44 (40–80)	52 (40–70)	0.927
*AVB I degree* (*N* = 182)	13 (71%)	0	6 (8%)	6 (14%)	1 (7%)	0.081
*Delta wave*	8 (4%)	1 (2%)	3 (4%)	1 (2%)	3 (20%)	0.016
*QRS interval(ms)*	91 (80–110)	82 (80–86)	92 (80–108)	105.5 (95–118)	130 (97–152)	<0.001
*QRS interval ≥ 120 ms*	27 (14%)	0	7 (9%)	11 (24%)	9 (60%)	<0.001
*RBBB, complete*	17 (9%)	0	3 (4%)	6 (13%)	8 (53%)	<0.001
*RBBB, incomplete*	25 (13%)	0	15 (20%)	10 (22%)	0	0.001
*Non-specific intraventricular conduction delay*	12 (6%)	1 (2%)	3 (4%)	6 (13%)	2 (13%)	0.07
*LAFB*	22 (12%)	0	6 (8%)	10 (22%)	6 (40%)	0.001
*Left axis deviation*	23 (12%)	0	6 (8%)	11 (24%)	6 (40%)	0.001
*QRS fragmentation*	24 (13%)	1 (2%)	8 (10%)	11 (24%)	4 (27%)	0.004
*QT interval (ms)*	396 (366–420)	380 (360–390)	392 (360–411)	402 (394–438)	434 (400–460)	<0.001
*QTcB interval (ms)*	416 (398–434)	412 (397–425)	413 (392–431)	422 (400–440)	452 (420–470)	<0.001
*Pathologic QTc interval*	9 (5%)	0	1 (1%)	1 (2%)	7 (47%)	0.001
*QTc interval ≥ 480 ms*	5 (23%)	0	1 (1%)	1 (2%)	3 (20%)	0.001
*Left atrial enlargement* (*N* = 182)	41 (22%)	2 (4%)	11 (1%)	19 (43%)	9 (64%)	<0.001
*Right atrial enlargement* (*N* = 182)	1 (1%)	0	0	1 (2%)	0	
*Cornell Voltage (ms)*	13 (9–20)	10 (7–12)	14 (10–20)	20 (12–26)	16 (12–25)	<0.001
*Positive Cornell Index*	24 (13%)	1 (2%)	7 (9%)	13 (28%)	3 (20%)	<0.001
*Sokolov Index (ms)*	25 (20–35)	22 (18–26)	27 (21–34)	31 (22–46)	30 (18–40)	<0.001
*Positive Sokolov Index (N = 187)*	47 (25%)	3 (6%)	18 (24%)	20 (44%)	6 (40%)	0.001
*Massive Sokolov Index*	16 (8%)	1 (2%)	5 (7%)	10 (22%)	0	0.002
*R wave aVL > 11 mm*	47 (25%)	2 (4%)	16 (21%)	20 (43%)	9 (60%)	<0.001
*LVH, at least one criterion* (*N* = 188)	75 (40%)	6 (11%)	29 (39%)	29 (63%)	11 (73%)	<0.001
*Total QRS score (ms)*	155 (122–212)	126 (105–150)	160 (125–198)	208 (161–269)	230 (150–320)	<0.001
*R wave V5 amplitude (mV)*	15 (11–21)	13 (10–16)	16 (12–25)	19 (12–25)	15 (11–25)	0.002
*Slurred QRS onset + borderline PR interval* (*N* = 178)	22 (12%)	5 (10%)	10 (14%)	6 (13%)	1 (9%)	0.9
*Pseudo-necrosis*	28 (15%)	3 (6%)	7 (9%)	15 (32%)	3 (20%)	0.001
*Inferior pseudonecrosis*	3 (2%)	0	0	3 (6%)	0	
*Anterior pseudonecrosis*	5 (3%)	0	0	3 (6%)	2 (13%)	0.005
*Lateral pseudonecrosis*	25 (13%)	3 (6%)	7 (9%)	13 (28%)	2 (13%)	0.006
*Poor precordial R wave progression*	12 (6%)	2 (4%)	3 (4%)	6 (13%)	1 (7%)	0.196
*LV repolarization abnormalities*	80 (42%)	5 (10%)	25 (33%)	36 (78%)	14 (94%)	<0.001
*Negative T waves*	71 (38%)	4 (8%)	20 (26%)	34 (74%)	13 (87%)	<0.001
*Inferior, symmetrical*	23 (12%)	2 (4%)	6 (8%)	9 (20%)	6 (40%)	<0.001
*Inferior, asymmetrical*	16 (8%)	2 (4%)	6 (8%)	5 (11%)	3 (20%)	<0.001
*Anterior, symmetrical*	15 (8%)	1 (2%)	3 (4%)	7 (15%)	4 (27%)	0.003
*Anterior, asymmetrical*	14 (7%)	1 (2%)	6 (8%)	5 (11%)	2 (13%)	0.003
*Lateral, symmetrical*	29 (15%)	2 (4%)	5 (7%)	16 (35%)	6 (40%)	<0.001
*Lateral, asymmetrical*	37 (20%)	1 (2%)	13 (17%)	16 (35%)	7 (47%)	<0.001
*Giant negative T wave*	13 (7%)	0	1 (1%)	7 (15%)	5 (33%)	<0.001
*ST-segment depression*	55 (29%)	4 (8%)	15 (20%)	28 (61%)	8 (53%)	<0.001
*Anterior*	5 (3%)	0	0	4 (9%)	1 (7%)	0.012
*Inferior*	19 (10%)	1 (2%)	6 (8%)	8 (17%)	4 (27%)	0.01
*Lateral*	50 (26%)	4 (8%)	13 (17%)	26 (56%)	7 (47%)	<0.001
*Giant positive T waves*	14 (7%)	0	5 (7%)	8 (17%)	1 (7%)	0.013
*Symmetrical (N = 181)*	11 (6%)	0	6 (8%)	5 (12%)	0	0.075
*Asymmetrical (N = 181)*	3 (3%)	0	0	2 (5%)	1 (8%)	0.062
*Anterior (N = 187)*	13 (7%)	0	5 (7%)	7 (16%)	1 (7%)	0.03
*Lateral (N = 187)*	4 (2%)	0	1 (1%)	3 (7%)	0	0.107
*Max T wave amplitude (mV)*	5 (4–8)	4 (3–7)	5 (4–8)	7 (4–10)	8 (4–10)	0.009
*ST-segment elevation*	25 (13%)	0	6 (8%)	15 (33%)	4 (27%)	<0.001
*Inferior*	5 (26%)	0	1 (1%)	3 (6%)	1 (7%)	0.138
*Anterior*	21 (11%)	0	6 (8%)	12 (26%)	3 (20%)	<0.001
*Lateral*	1 (1%)	0	0	1 (2%)	0	

AVB, atrioventricular block; LAFB, left anterior fascicular block; LV, left ventricular; LVH, left ventricular hypertrophy; RBBB, right bundle branch block

### 4.1. Group analysis

A normal ECG was present in 87% of patients in group A (LVWT ≤ 9 mm), 54% in group B (LVWT 10–14 mm), 11% in group C (LVWT 15–19 mm), and 7% in group D (LVWT ≥ 29 mm), respectively (*p* < 0.001). All patients from group A were on sinus rhythm, and atrial fibrillation was more prevalent in groups C and D (7% and 13% respectively). Regarding atrio-ventricular conduction, PR shortening (<120 ms) was more frequent in group A (13%), whereas with LVWT increase median PR interval duration showed a progressive prolongation among the groups (137 [125–154] vs. 140 [127–160] vs. 160 [130–180] vs. 170 [130–180] ms respectively, *p* = 0.002). No differences were noted between the presence of a borderline PR interval (120–130 ms) and the increase in LVWT. Median P wave duration in lead DII was significantly shorter in groups A and B compared to groups C and D (80 ms vs. 100 ms, *p* < 0.001), while P wave end-to-Q wave interval in DII was not significantly affected by the degree of LVH (*p* = 0.927). QRS interval duration gradually increased across the groups, with 24% of patients from in group C and 60% of patients from in group D showing a QRS ≥ 120 ms (*p* < 0.001). RBBB presence significantly increased across the study subgroups (*p* < 0.001); no cases of RBBB were noted in group A, incomplete RBBB was observed predominantly in groups B and C (respectively 20% and 22%), while patients from group D exhibited exclusively complete RBBB in group D (54%). None of the patients had LBBB. As LVH increased, LAFB (0%, 8%, 22%, 40%, *p* < 0.001) and QRS fragmentation (2%, 11%, 25%, 23%, *p* = 0.009) had higher prevalence. Corrected QT interval gradually increased among groups (*p* < 0.001), and 20% of patients from group D had QTc ≥ 480 ms (*p* = 0.01). No differences were found regarding LV pre-excitation prevalence across the groups; no patient in the entire population showed low QRS voltages. As expected, all ECG criteria for LVH evaluated in the study (Cornell index, Sokolow-Lyon index, R wave amplitude in aVL) statistically augmented among the groups (*p* < 0.001), along with total QRS score (126 [105–150] vs. 160 [125–198] vs. 208 [161–269] vs. 230 [150–320] mm, *p* < 0.001). Similarly, right ventricular hypertrophy and left atrial enlargement were more common in patients with higher degree of LVH (*p* = 0.02 and *p* < 0.001 respectively). Group C and D showed the highest prevalence of pseudonecrosis (respectively 32% and 20%, *p* < 0.001), mostly in anterior and lateral leads. In line with LVWT increase, a higher prevalence of LV repolarization abnormalities was observed: negative T waves were more frequent in group D (87%, *p* < 0.001), and involved mostly lateral and inferior leads. Giant negative T waves were present only in groups C and D (15% and 33% respectively); they were mostly symmetrical and involving the anterior or lateral leads (Table 2). Likewise, ST segment depression was more common in inferior and lateral leads. Only patients with LVH (LVWT ≥ 10 mm) showed giant positive T waves (7%, 17%, 7% of patients from group B, C and D respectively *p* = 0.013), involving mostly anterior leads. Among all groups, maximum T wave amplitude was higher in patients from group D (8 mm, *p* < 0.001).

### 4.2. ECG patterns

**Figure 1:**
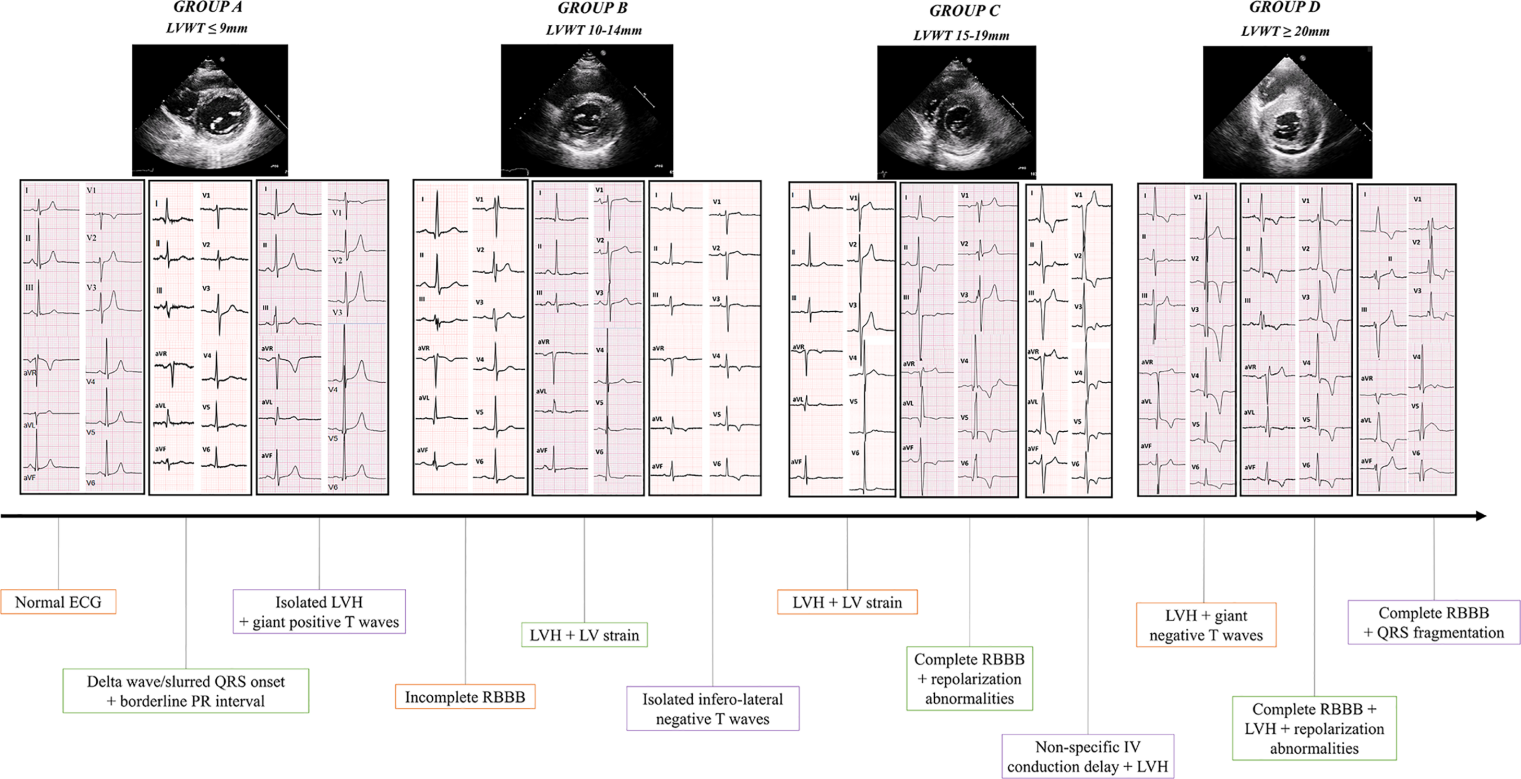
Central figure. The different ECG patterns representative of each AFD stage according to the LVWT are shown. Descriptions are listed form left to right. Group **A** (LVWT ≤ 9 mm, 28% of the population): normal ECG; delta wave/slurred QRS onset + borderline PR interval; isolated LVH + giant positive T waves. Group **B** (LVWT 10–14 mm, 40% of the cohort): incomplete RBBB; LVH + LV “strain”; isolated infero-lateral negative T waves. Group **C** (LVWT 15–19 mm, 24% of the population): LVH + LV “strain”; complete RBBB + repolarization abnormalities; non-specific intraventricular conduction delay + LVH. Group **D** (LVWT ≥ 20 mm, 8% of the cohort): LVH + giant negative T waves; complete RBBB + LVH + repolarization abnormalities; complete RBBB + QRS fragmentation. AFD, anderson-fabry Disease; LVH, left ventricular hypertrophy; LVWT, left ventricular wall thickness; RBBB, right bundle branch block.

**Figure 2:**
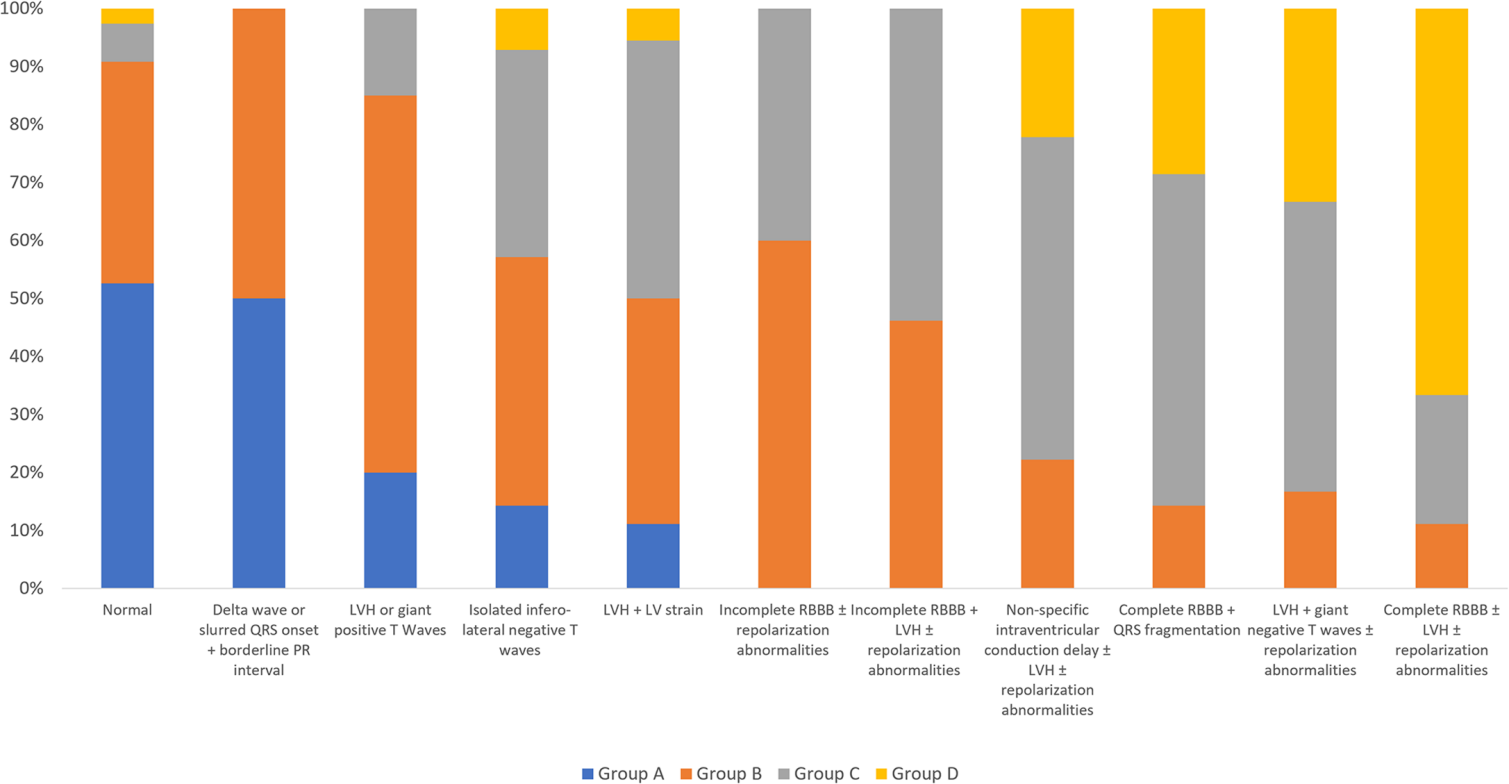
Histogram of the ECG patterns and their prevalence according to the study groups. Patients are classified into one exclusive ECG pattern. LV, left ventricular; LVH, left ventricular hypertrophy; RBBB, right bundle branch block.

As an additional analysis, we evaluated the presence of peculiar ECG characteristics within the study population, in a view to detect patterns suggestive of the different AFD cardiac stages, and thus to describe the progression of LVH along the natural history of the disease (Figures 1, 2). In this view, an important result from this cohort is that only 13% of the patients with normal wall thickness (group A) showed ECG abnormalities, which were quite atypical and represented mainly by isolated negative T waves in infero-lateral leads (4%), LVH criteria/giant positive T waves (8%), and delta wave/slurred QR onset together with borderline PR (8%). Differently, patients with intermediate phenotypic AFD expression (LVWT between 10 and 14 mm in group B and 15–19 mm in group C, respectively), showed more heterogeneous electrocardiographic alterations: LVH/giant positive T waves (17% and 7% of patients from group B and C, respectively); LVH + LV strain (9% and 17% from group B and C, respectively); incomplete RBBB associated with repolarization abnormalities (8% and 9% of patients in groups B and C, respectively) which were more frequently associated with LVH criteria in group C rather than group B (8% and 15%, respectively). At the other extreme of the spectrum, in patients from group D (LVWT ≥ 20 mm, group D) ECG was profoundly altered, being the most frequent example represented by complete RBBB associated to LVH criteria and repolarization abnormalities (40%), sometimes with QRS fragmentation (13%).

## 5. Discussion

### 5.1. P wave and PR interval

PR interval is frequently altered in AFD, and PR shortening can be detected in the initial phase of the disease, even with no LVH (12). Consistent with these previous observations, in our cohort 13% of patients from group A (no LVH), 10% from group B (LVWT 10–14), and 5% from group C (LVWT 15–19) had a short PR interval (<120 ms), whereas no patients with severe LVH (LVWT ≥ 20 mm, group D) showed this alteration. Another remarkable result in our study is the association of a borderline PR interval (120–130 ms) with a slurred QRS onset in patients LVWT ≥ 10 mm (14% of patients from both groups B and C). This specific ECG pattern is considered a consequence of the increased atrio-ventricular conduction velocity caused by intracellular glycosphingolipids, without evidence of an accessory pathway: Birket et al. (13) demonstrated enhanced sodium channel function, higher spontaneous action potentials frequency and shorter action potentials in AFD patient-derived induced pluripotent stem cells, with a higher cellular excitability potentially responsible for these alterations. This could be the reason of the gradual PR interval duration increase in line with the progressive LVH and the severity of the disease observed in our cohort. Specifically, and according to our results, PR interval increase is mostly due to the progressive P wave duration augmentation along with LVWT, and P wave duration has higher specificity than PR interval (12). Recently, Augusto et al. (14) observed an interesting biphasic trend of P wave duration during the course of AFD: at the very beginning, during the pre-hypertrophic phase, P wave had a shorter duration, reflecting the elevated intra-atrial conduction velocity, while with the progressive Gb3 accumulation and the consequent atrial remodeling, a pseudo-normalization first and finally a prolongation in P wave duration could be observed. In line with these studies, Zada et al. (15) have shown a statistically significant correlation between left atrial volume indexed and PQ interval in their 45 cohort of genetically proved AFD patients. P wave shortening at the initial stages of the disease may be the result of a coordinated and synchronous instead of sequential bi-atrial depolarization, due to the sinus node activity preferentially exiting closer to Bachmann's bundle. Enzymatic replacement therapy could interrupt this process and restore the physiologic atrial activation with a consequent increase in P wave duration, which thus represents a valuable marker in clinical management of AFD patients. Therefore, according to other studies in this field (5, 14), we evaluated P wave end to QRS onset duration, being this parameter not influenced by modifications in P wave duration, as well as we did not observe significant changes among the different LVH degree (*p* = 0.9).

### 5.2. Bradi- and tachyarrhythmias

In addition to morphologic and functional myocardial impairment, AFD also affects the cardiac conduction system at all levels, from the sinoatrial node to the distal ramification of Purkinje fibers (16) In a cohort of 53 AFD patients analyzed by Di et al, bradyarrhythmias were observed as a common manifestation of cardiac involvement (17) The authors found that age, LV mass, LV ejection fraction and LA dysfunction (defined as lower maximal peak positive strain at echocardiography) were markers associated with bradyarrhythmias. The mechanisms of bradyarrhythmia in AFD are not completely understood, but histological studies described fibrosis and apoptosis of cardiac conduction tissue as frequent findings in post-mortem analysis (18–21). Our results are in line with these reports, and even if the evaluation of cardiovascular events are not specifically assessed in the study, patients with indications for pacemaker implantation were mostly from group D (20% of this group), in which higher left atrial diameters were observed (median antero-posterior diameter in parasternal long axis view of 48.5 mm, *p* < 0.001). In accordance with the study by Shah et al. (22), in which 4% of AFD patients were diagnosed with atrial fibrillation during a median follow up of 1.9 years, we observed a global prevalence of atrial fibrillation of 5%, higher in patients from groups C and D, even if not statistically associated with LVWT (7% and 13% respectively, *p* = 0.364). No patients without LVH (group A) showed this arrhythmia in our population, differently from the literature where have been described cases of lone atrial fibrillation as first clinical manifestation in AFD patients with normal LV wall thickness (23). Many factors may be linked to atrial fibrillation in AFD, such as progressive Gb3 and lyso-Gb3 accumulation, atrial remodeling, and diastolic dysfunction. Indeed, atrial fibrillation is seldom observed young adults, being an intermediate-to-late disease complication (22).

### 5.3. Left ventricular hypertrophy

In line with the current literature, which reports LVH as the commonest structural abnormality in AFD patients (24), 72% of our population showed LVH, defined in our study as LVWT ≥ 10 mm. This is caused by intra-cellular Gb3 accumulation, by hypertrophy-inducing growth factors releasing, and by extracellular matrix remodeling. Experimental studies demonstrated that, when compared to normal controls of hypertensive population, plasma of AFD patients induces rat vascular smooth muscle cells and cardiomyocytes proliferation in culture (25). Other studies identified a proliferative factor in plasma of AFD patients, sphingosine-1 phosphate (S1P), and observed that its levels correlated with LV mass index, being this molecule capable of inducing cardiac hypertrophy S1P-treated mice (26). In our study, ECG abnormalities indicative of LVH such as Sokolow-Lyon and Cornell index can be observed in all the 3 groups with LVWT ≥ 10 mm, with higher frequencies as the hypertrophy increased (21%, 44% and 60% of patients from groups B, C, and D respectively, *p* < 0.001) As previously reported, we observed a positive Sokolow-Lyon index also in 12% of patients with no echocardiographic LVH, indicating that ECG changes may precede cardiac imaging abnormalities (27). QRS total score, the algebraic sum of zenith-to-nadir QRS amplitudes of all 12 leads, correlates with LV mass with evidence of a higher sensibility for LVH detecting compared to other ECG criteria (28). We observed a progressive QRS total score increase in line with LVH expression, with a QRS total score > 175 mm in all patients with LVWT ≥ 15 mm. Another ECG sign associated with LVH is represented by left atrial enlargement, which was statistically related to LVWT increase (*p* < 0.001). This could be the result of LV stiffness increase along with cardiac hypertrophy progression, leading to higher end-diastolic left ventricular and atrial pressure.

### 5.4. Intraventricular conduction: QRS interval

Similar to PR interval, QRS alterations in AFD are related to the progressive glycosphingolipids accumulation and follow a biphasic pattern. During the initial phase of the disease, acceleration of intra-ventricular conduction and QRS narrowing could be observed, whereas in later stages with cellular hypertrophy and interstitial fibrosis a progressive degeneration of myocardial conduction system can be detected, leading to QRS prolongation (5, 12). Indeed, we reported a significant association between QRS duration and LVH degree, so that 24% of patients from group C (LVWT 15–19 mm) and more than half patients (60%) from group D (LVWT ≥ 20 mm) had a QRS interval ≥ 120 ms (*p* < 0.001). In particular, 20% and 22% of patients from group B and C respectively showed incomplete RBBB, whereas patients from group D had only complete RBBB (53%). The appearance of an atypical/incomplete RBBB rather than a classical complete one may be explained by the coexistence of different degrees of LVH intertwined with the conduction delay hallmarks (rsR' or pR), and with the progressive increase of Gb3 accumulation, which changes the myocardial substrate both at truncular and peripheral (Purkinje fibers) level. An important result is that none of the patients from our cohort had LBBB. Patients with severe LVH frequently exhibited LAFB (40%), constantly associated with complete or incomplete RBBB (bi-fascicular block). Other studies demonstrated RBBB more commonly than LBBB in AFD patients: the reasons of the peculiar susceptibility of the right bundle branch are not completely clear, but could be explained by several factors, namely the thinner anatomical structure of the RBB compared to the thicker and broader posterior fascicle of the LBB, its superficial course at the right ventricular septal prospect, and a more prevalent early Gb3 accumulation at the basal interventricular septum level (7, 29). Additionally, we reported a higher frequency of QRS fragmentation in line with LVH extent (27% of group D patients), presumably due to the presence of intra-cardiac fibrosis in the final stage of AFD and consequently to the potential conduction slowing (30).

### 5.5. LV repolarization abnormalities

**Figure 3:**
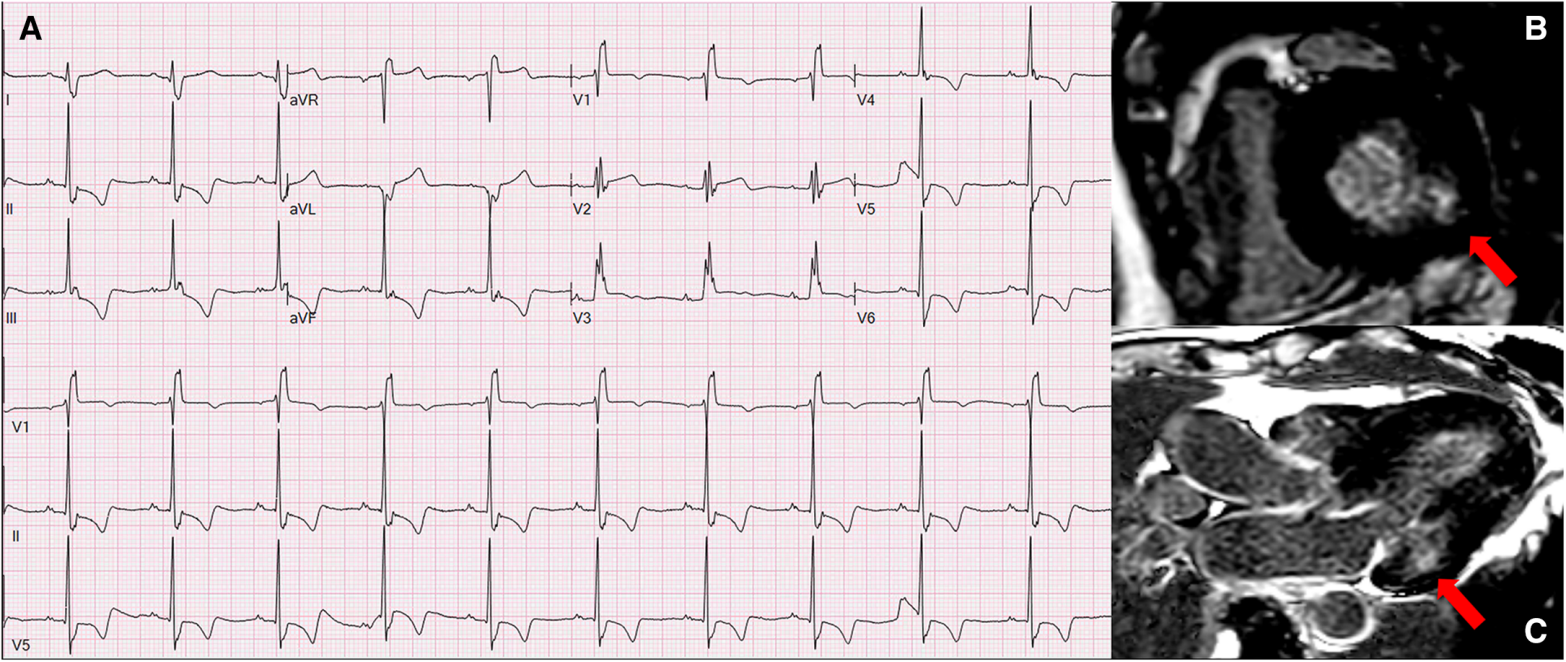
Panel A represents the ECG of 70-year-old women from group C (LVWT = 15 mm) with a late onset AFD phenotype and shows normal sinus rhythm at 70 bpm, with left atrial enlargement, complete RBBB, and infero-lateral negative T waves, which correspond to the basal infero-lateral LGE distribution at the CMR (panels B and C). AFD, anderson-fabry disease; CMR, cardiac magnetic resonance; LGE, late gadolinium enhancement; RBBB, right bundle branch block.

In AFD, ECG abnormalities also involve LV repolarization, and many studies in this field reported ST segment and T wave alterations as frequent findings in these patients (8, 31). We observed a significant association between the presence of LV repolarization abnormalities and LVH progression (*p* = 0.001). The most frequent alterations were negative T waves, noted in 74% and 87% of patients with LVWT ≥ 15 mm, mainly in lateral and inferior leads. Remarkably, 33% of patients from group D (LVWT ≥ 20 mm) showed giant negative T waves (amplitude ≥ 1 mV), which may resemble the LVH medium-apical distribution at the echocardiogram. ST segment depression was typically associated with T waves inversion, involved mostly inferior and lateral leads, and was significantly more prevalent along as LVH increased (*p* = 0.001). LV repolarization abnormalities are likely secondary to LVH, but several evidence reported an association with late gadolinium enhancement distribution at cardiac magnetic resonance, which involves predominantly the LV basal infero-lateral segments and ECG abnormalities localization (V5, V6, DIII, aVF) (Figure 3) (32, 33). In our cohort we noticed a similar prevalence of asymmetrical negative T waves, usually thought to be secondary to LVH, and symmetrical ones, which are more suggestive for the presence of late gadolinium enhancement (inferior 8% and 12%, anterior 7% and 8%, lateral 20% and 15% respectively). Niemann et al. suggested that the absence of ST segment or T wave alterations on electrocardiogram could almost exclude LGE presence at CMR (7); however, LGE has been recently described in up to 18% of the patients without ST depression and 13% of the patients without negative T waves in a large cohort of patients with late-onset FD with predominant cardiac involvement (34). In addition, 34% and 27% of patients from groups C and D showed ST segment elevation, mostly in anterior leads. Cardiomyocyte hypertrophy, as well as transmembrane ion pumps, interstitial fibrosis, and inflammation, are associated with QT and QTc prolongation (12), which we noted to be significantly more pronounced in line with LVH progression (47% of patients with LVWT ≥ 20 mm).

Of course, arterial hypertension is associated with electrocardiographic alterations, mostly in terms of LVH signs; however, as observed in a recent relevant systematic review, when all the ECG parameters were evaluated, only few were found to be consistently and significantly associated with blood pressure values, represented by P wave dispersion, TpTe interval and QTc interval (35). In our cohort arterial hypertension is more frequent in groups C and D, but in addition to the expected ECG abnormalities such as the prolonged QTc interval and the P wave enlargement, we found other remarkable changes, represented by the higher prevalence of right bundle branch block and QRS fragmentation. By the note, none of our patients exhibit left bundle branch block, which is frequently observed in patients with arterial hypertension and other age-related cardiovascular comorbidities, such as valvular diseases and in particular aortic stenosis (36).

### 5.6. ECG patterns

Standard ECG analysis through a systematic methodology and interpretation of the abnormalities with a “cardiomyopathy-oriented” approach play a pivotal role in the diagnosis and management of cardiomyopathies (37). In the setting of patients with hypertrophic phenotype, the ECG is able to predict the possibility of a phenocopy and can guide the clinician in the request of second/third level exams needed to achieve the specific diagnosis (38).

Recently, El Sayed et al. published an interesting longitudinal cohort study of a large cohort of AFD patients, describing the evolution of ECG alterations during a follow up of 20 years. Many of the evaluated ECG parameters showed progressive and significant changes over time, with P-wave and PR interval increase, QRS and QTc interval prolongation, increase of LVH indexes with increasing age. Considering that the age reflects the longer course of the disease and the progressive accumulation of glycosphingolipids (expressed in our study by the wall thickness increase), we observed similar results, with patients from group C and D older and with more pronounced ECG abnormalities than patients from groups A and B. Moreover, El Sayed et al. performed an interesting comparison between AFD patients and a matched healthy subjects' cohort, showing that frontal QRS-axis is the earliest marker of cardiac disease being already significant in both male and female AFD patients aged 18–29 years. On the other hand, in addition to the description of the parameters changes over time, we provide distinct ECG patterns for each of the study group, which are able to describe the progressive cardiac involvement by the disease (39).

A previous study from our group (9) defined an easily applicable ECG-based score for patients with unexplained LV hypertrophy, able to distinguish between sarcomeric HCM and AFD, with a good performance. In this view, the different ECG patterns proposed in this study (Figures 1, 2) may act not only as red flags for AFD suspicion in the context of hypertrophic cardiomyopathies' phenotypes, but also and, more importantly, to track the progression of the disease, starting from the pre-hypertrophic phase, going through the mild-to-moderate disease expression in terms of LVH, and finally reaching the final stage with severe hypertrophy degree. In other words, ECG presents itself as the storyteller for patients with AFD.

## 6. Study limits

Being retrospective and multicenter, our study has some limitations. Sample size of groups C and D (LVWT ≥ 15 mm) is relatively small. In addition, more than half of the patients with AFD were already on specific therapy, whose effects on the ECG are unknown. LV strain and diastolic function parameters, as well as the degree of valvular disease, were not reported because a full echocardiographic assessment was not the study purpose. Finally, considering the heterogeneity in terms of disease burden before starting AFD specific therapy and therapy duration, comparisons between untreated and treated patients were not made.

## 7. Conclusions

In the present study we describe ECG abnormalities of AFD and more specifically we provide ECG patterns associated with different stages of the disease, stratifying the population according to the severity of LVH, which is the echocardiographic manifestation of the progressive accumulation of glycosphingolipids over time. These ECG patterns represent “instantaneous pictures” along of the natural history of AFD. In patients with no LVH (pre-hypertrophy stage) some clues of cardiologic cardiac involvement may be identified at ECG analysis, such as short/borderline PR interval or electrocardiographic signs of LVH in a small percentage of subjects. As LVWT wall thickness increases, LVH criteria, intraventricular conduction delay (mainly RBBB) and repolarization abnormalities in terms of negative T waves or ST segment elevation/depression become more frequent. Finally, in the last stage of the disease, the main ECG findings were represented by complete RBBB and LVH, QRS fragmentation, or giant negative T waves when LVH distribution was mid-apical. Whether ECG changes may be associated with clinical events remains to be determined; however, our study represents the first step for further analysis in risk stratification for major adverse events based on such an easy tool.

## Data Availability

The original contributions presented in the study are included in the article, further inquiries can be directed to the corresponding author.

## References

[1] Brady RO Gal AE Bradley RM Martensson E Warshaw AL Laster L. Enzymatic defect in Fabry's Disease. N Engl J Med (2010) 276:1163–7. 10.1056/NEJM196705252762101 6023233

[2] Zarate YA Hopkin RJ. Fabry's disease. Lancet. (2008) 372:1427–35. 10.1016/S0140-6736(08)61589-5 18940466

[3] Pieroni M Moon JC Arbustini E Barriales-Villa R Camporeale A Vujkovac AC Cardiac involvement in fabry disease: jACC review topic of the week. J Am Coll Cardiol. (2021) 77:922–36. 10.1016/j.jacc.2020.12.024 33602475

[4] Mehta A Clarke JTR Giugliani R Elliott P Linhart A Beck M Natural course of fabry disease: changing pattern of causes of death in FOS—fabry outcome survey. J Med Genet. (2009) 46:548–52. 10.1136/jmg.2008.065904 19473999

[5] Namdar M Steffel J Vidovic M Brunckhorst CB Holzmeister J Lüscher TF Electrocardiographic changes in early recognition of fabry disease. Heart (2010) 97:485–90. 10.1136/hrt.2010.211789 21270075

[6] Schmied C Nowak A Gruner C Olinger E Debaix H Brauchlin A The value of ECG parameters as markers of treatment response in fabry cardiomyopathy. Heart. (2016) 102:1309–14. 10.1136/heartjnl-2015-308897 27056970

[7] Niemann M Hartmann T Namdar M Breunig F Beer M Machann W Cross-sectional baseline analysis of electrocardiography in a large cohort of patients with untreated fabry disease. J Inherit Metab Dis. (2013) 36:873–9. 10.1007/S10545-012-9540-8 23053470

[8] Figliozzi S Camporeale A Boveri S Pieruzzi F Pieroni M Lusardi P ECG-based score estimates the probability to detect fabry disease cardiac involvement. Int J Cardiol. (2021) 339:110–7. 10.1016/J.IJCARD.2021.07.022 34274410

[9] Vitale G Ditaranto R Graziani F Tanini I Camporeale A Lillo R Standard ECG for differential diagnosis between anderson-fabry disease and hypertrophic cardiomyopathy. Heart. (2022) 108:54–60. 10.1136/heartjnl-2020-318271 33563631

[10] Zamorano JL Anastasakis A Borger MA Borggrefe M Cecchi F Charron P 2014 ESC guidelines on diagnosis and management of hypertrophic cardiomyopathy: the task force for the diagnosis and management of hypertrophic cardiomyopathy of the European society of cardiology (ESC). Eur Heart J. (2014) 35:2733–79. 10.1093/eurheartj/ehu284 25173338

[11] Strauss DG Selvester RH Wagner GS. Defining left bundle branch block in the era of cardiac resynchronization therapy. Am J Cardiol. (2011) 107:927–34. 10.1016/J.AMJCARD.2010.11.010 21376930

[12] Namdar M. Electrocardiographic changes and arrhythmia in fabry disease. Front Cardiovasc Med. (2016) 3:1–6. 10.3389/fcvm.2016.00007 PMC4805598 27047943

[13] Birket MJ Raibaud S Lettieri M Adamson AD Letang V Cervello P A human stem cell model of fabry disease implicates LIMP-2 accumulation in cardiomyocyte pathology. Stem Cell Rep. (2019) 13:380–93. 10.1016/J.STEMCR.2019.07.004 PMC6700557 31378672

[14] Augusto ao B Johner N Shah D Nordin S Knott KD Rosmini S The myocardial phenotype of fabry disease pre-hypertrophy and pre-detectable storage. Eur Heart J Cardiovasc Imaging (2021) 22(7):790–99. 10.1093/ehjci/jeaa101 32514567 PMC8219366

[15] Zada M Lo Q Trivedi SJ Harapoz M Boyd AC Devine K Electrocardiographic characteristics and their correlation with echocardiographic alterations in fabry disease. J Cardiovasc Dev Dis. (2022) 9:11. 10.3390/jcdd9010011 35050221 PMC8777656

[16] Frustaci A Morgante E Russo MA Scopelliti F Grande C Verardo R Pathology and function of conduction tissue in fabry disease cardiomyopathy. Circ Arrhythm Electrophysiol. (2015) 8:799–805. 10.1161/CIRCEP.114.002569 26047621

[17] Di LZ Pichette M Nadeau R Bichet DG Poulin F. Severe bradyarrhythmia linked to left atrial dysfunction in fabry disease-A cross-sectional study. Clin Cardiol. (2018) 41:1207–13. 10.1002/CLC.23019 29959806 PMC6490062

[18] Linhart A Elliott PM. The heart in anderson-fabry disease and other lysosomal storage disorders. Heart. (2007) 93:528–35. 10.1136/HRT.2005.063818 17401074 PMC1861503

[19] Sheppard MN Cane P Florio R Kavantzas N Close L Shah J A detailed pathologic examination of heart tissue from three older patients with Anderson-Fabry disease on enzyme replacement therapy. Cardiovasc Pathol. (2010) 19:293–301. 10.1016/J.CARPATH.2009.05.003 19631563

[20] Ikari Y Kuwako K Yamaguchi T. Fabry's disease with complete atrioventricular block: histological evidence of involvement of the conduction system. Br Heart J. (1992) 68:323–5. 10.1136/HRT.68.9.323 1389767 PMC1025080

[21] Frustaci A Chimenti C. Cryptogenic ventricular arrhythmias and sudden death by fabry disease: prominent infiltration of cardiac conduction tissue. Circulation. (2007) 116. 10.1161/CIRCULATIONAHA.107.723387 17875975

[22] Shah JS Hughes DA Sachdev B Tome M Ward D Lee P Prevalence and clinical significance of cardiac arrhythmia in anderson-fabry disease. Am J Cardiol. (2005) 96:842–6. 10.1016/J.AMJCARD.2005.05.033 16169374

[23] Chimenti C Russo MA Frustaci A. Atrial biopsy evidence of fabry disease causing lone atrial fibrillation. Heart. (2010) 96:1782–3. 10.1136/HRT.2010.196162 20736197

[24] Linhart A Kampmann C Zamorano JL Sunder-Plassmann G Beck M Mehta A Cardiac manifestations of Anderson–Fabry disease: results from the international fabry outcome survey. Eur Heart J. (2007) 28:1228–35. 10.1093/EURHEARTJ/EHM153 17483538

[25] Barbey F Brakch N Linhart A Rosenblatt-Velin N Jeanrenaud X Qanadli S Cardiac and vascular hypertrophy in fabry disease evidence for a new mechanism independent of blood pressure and glycosphingolipid deposition. Arterioscler Thromb Vasc Biol. (2006) 26:839–44. 10.1161/01.ATV.0000209649.60409.38 16469946

[26] Brakch N Dormond O Bekri S Golshayan D Correvon M Mazzolai L Evidence for a role of sphingosine-1 phosphate in cardiovascular remodelling in fabry disease. Eur Heart J. (2010) 31:67–76. 10.1093/EURHEARTJ/EHP387 19773225

[27] Linhart A Paleček T Bultas J Ferguson JJ Hrudová J Karetová D New insights in cardiac structural changes in patients with Fabry's Disease. Am Heart J. (2000) 139:1101–8. 10.1067/MHJ.2000.105105 10827394

[28] Dollar AL Roberts WC. Usefulness of total 12-lead qrs voltage compared with other criteria for determining left ventricular hypertrophy in hypertrophic cardiomyopathy: analysis of 57 patients studied at necropsy. Am J Med. (1989) 87:377–81. 10.1016/S0002-9343(89)80817-4 2529761

[29] Akhtar MM Elliott PM. Anderson-Fabry disease in heart failure. Biophys Rev. (2018) 10:1107–19. 10.1007/s12551-018-0432-5 29909504 PMC6082315

[30] Pietrasik G Zareba W. QRS Fragmentation: diagnostic and prognostic significance. Cardiol J. (2012) 19:114–21. 10.5603/CJ.2012.0022 22461043

[31] Sachdev B Elliott PM. Isolated cardiac manifestations in fabry disease: the UK experience. Acta Paediatr Supp. (2002) 91:28–30. 10.1111/J.1651-2227.2002.TB03106.X 12572839

[32] O’mahony C Elliott P. Anderson-Fabry disease and the heart. Prog Cardiovasc Dis. (2010) 52:326–35. 10.1016/j.pcad.2009.11.002 20109602

[33] Moon JCC Sachdev B Elkington AG McKenna WJ Mehta A Pennell DJ Gadolinium enhanced cardiovascular magnetic resonance in Anderson-Fabry disease. Evidence for a disease specific abnormality of the myocardial interstitium. Eur Heart J. (2003) 24:2151–5. 10.1016/J.EHJ.2003.09.017 14643276

[34] Azevedo O Gago MF Miltenberger-Miltenyi G Robles AR Costa MA Pereira O Natural history of the late-onset phenotype of fabry disease due to the p.F113l mutation. Mol Genet Metab Rep. (2020) 22:100565. 10.1016/J.YMGMR.2020.100565 32099817 PMC7026617

[35] Bird K Chan G Lu H Greeff H Allen J Abbott D Assessment of hypertension using clinical electrocardiogram features: a first-ever review. Front Med. (2020) 7:797. 10.3389/FMED.2020.583331/BIBTEX PMC7746856 33344473

[36] Pérez-Riera AR Barbosa-Barros R de Rezende Barbosa MPC Daminello-Raimundo R de Abreu LC Nikus K. Left bundle branch block: epidemiology, etiology, anatomic features, electrovectorcardiography, and classification proposal. Ann Noninvasive Electrocardiol. (2019) 24(2). 10.1111/ANEC.12572 PMC6931474 29932265

[37] Rapezzi C Arbustini E Caforio ALP Charron P Gimeno-Blanes J Heliö T Diagnostic work-up in cardiomyopathies: bridging the gap between clinical phenotypes and final diagnosis. A position statement from the ESC working group on myocardial and pericardial diseases. Eur Heart J. (2013) 34:1448–58. 10.1093/eurheartj/ehs397 23211230

[38] Finocchiaro G Sheikh N Biagini E Papadakis M Maurizi N Sinagra G The electrocardiogram in the diagnosis and management of patients with hypertrophic cardiomyopathy. Heart Rhythm. (2020) 17:142–51. 10.1016/j.hrthm.2019.07.019 31349064

[39] Sayed M E Postema PG Datema M van Dussen L Kors JA ter Haar CC ECG Changes during adult life in fabry disease: results from a large longitudinal cohort study. Diagnostics. (2023) 13:354. 10.3390/DIAGNOSTICS13030354 36766461 PMC9913957

